# Supplementary material for: Recruiting Perovskites to Degrade Toxic Trinitrotoluene
Source: Materials (Basel). 2021 Dec 2;14(23):7387. doi: 10.3390/ma14237387 (PMC8658843; doi:10.3390/ma14237387)
Supplement: Supplementary file 1 [file materials-14-07387-s001.zip › materials-1451933-SI.pdf]

materials

*Supporting Information*

# Recruiting Perovskites to Degrade Toxic Trinitrotoluene

**Yuri A. Mastrikov, Roman Tsyshevsky, Fenggong Wang and Maija M. Kuklja ***

Department of Materials Science and Engineering, University of Maryland, College Park 20742, MD, USA; yuri@umd.edu (Y.A.M.); rtsyshev@umd.edu (R.T.); fenggong.wang@gmail.com (F.W.)
* Correspondence: mkukla@nsf.gov

**Citation:** Mastrikov, Y.A.; Tsyshevsky, R.; Wang, F.; Kuklja, M.M. Recruiting Perovskites to Degrade Toxic Trinitrotoluene. *Materials* **2021**, *14*, 7387. https://doi.org/10.3390/ma14237387

Academic Editor: Klaus Stowe

Received: 21 October 2021
Accepted: 27 November 2021
Published: 2 December 2021

**Publisher's Note:** MDPI stays neutral with regard to jurisdictional claims in published maps and institutional affiliations.

**Copyright:** © 2021 by the authors. Submitted for possible open access publication under the terms and conditions of the Creative Commons Attribution (CC BY) license (http://creativecommons.org/licenses/by/4.0/).

**Table S1.** Calculated lattice parameters of BTO, BSTO, and STO ideal bulk crystals in *R*3*m* space group (α=90°) and the (001) $4a_0 \times 4a_0$ surface cell 7-plane slab with the $5a_0$-wide vacuum gap.

| Lattice parameter | Material | BTO | BSTO | STO |
|---|---|---|---|---|
| $a_0$, Å | bulk | 4.05 (4.00 [a]) | 4.01 | 3.95 (3.90 [b]) |
| | surface | 4.03 | 3.97 | 3.92 |

[a] Experimental lattice constants of BTO were taken from ref.[1].

[b] Experimental lattice constants of STO were taken from ref.[2].
